# GDC-0449 improves the antitumor activity of nano-doxorubicin in pancreatic cancer in a fibroblast-enriched microenvironment

**DOI:** 10.1038/s41598-017-13869-0

**Published:** 2017-10-17

**Authors:** Quan Zhou, Yongcun Zhou, Xiangrui Liu, Youqing Shen

**Affiliations:** Key Laboratory of Biomass Chemical Engineering of Ministry of Education and Center for Bionanoengineering, College of Chemical and Biological Engineering, Zhejiang University, Hangzhou, China

## Abstract

Pancreatic cancer is one of the most lethal human cancers that currently does not have effective therapies. Novel treatments including nanomedicines and combination therapies are thus urgently needed for these types of deadly diseases. A key feature of pancreatic cancer is its tumor protective dense stroma, which is generated by cancer-associated fibroblasts (CAFs). The interaction between CAFs and pancreatic cancer cells abnormally activates sonic hedgehog (SHH) signaling and facilitates tumor growth, metastasis, and drug resistance. Here, we report that the commercial SHH inhibitor GDC-0449 reverses fibroblast-induced resistance to doxorubicin in Smoothened (SMO)-positive pancreatic cancer cells by downregulating SHH signaling proteins. Importantly, the synergistic combination of GDC-0449 with PEG-PCL-Dox exhibited potent antitumor efficacy in a BxPC-3 tumor xenograft model, whereas single treatments did not significantly inhibit tumor growth. Our findings reveal a potential treatment strategy for fibroblast-enriched pancreatic cancer.

## Introduction

The enhanced permeability and retention (EPR) effect was first reported by Dr. Hiroshi Maeda in 1986, and its increased accumulation in tumor tissues and reduced system toxicity supports the benefits of nanodrugs over small molecules^1,2^. Since then, countless nanocarriers for cancer-targeted drug delivery have been developed in the past 30 years, and several are currently commercially available for cancer therapy. However, clinical feedback indicates that the improvements of nano-sized anticancer formulations are limited, particularly in terms of therapeutic efficacy^3^. In recent years, the roles of fibroblasts in the tumor microenvironment have been investigated, and growing evidence indicates that fibroblasts not only restrain drug sensitivity but also limit the penetration of drugs in tumors^4^. However, previous research studies on drug discovery have mainly focused on cancer cells, whereas investigations on the influence of stromal fibroblasts on drug response are limited^5^.

Pancreatic cancer is one of the most devastating human malignancies with a 5-year survival rate of <5%^5–7^. Novel combination therapies targeting different signaling pathways are thus needed to combat this lethal disease. Aberrant activation of the sonic hedgehog (SHH) signaling has been widely observed in pancreatic cancer^8,9^. As a mediator of tumorigenesis, paracrine SHH signaling plays an important role in the communication between tumor and activated fibroblasts^10,11^. SHH ligands secreted by tumor cells positively regulate signaling in the surrounding tumor stroma, whereas stroma fibroblast cells can also serve as a resource for SHH ligands, which then activate SHH signaling in tumor cells through an inverse paracrine way^12^. SHH ligands bind to the Patched protein, which thereby releases inhibition of transducer protein Smoothened (SMO) and then triggers GLI transcription factors, ultimately resulting in the induction of downstream target genes^13^. The downstream genes activated by GLI include the *Gli1* and *Patched* genes that upregulate tumor cell growth, drug resistance, and epithelial-mesenchymal transition (EMT)^14^. Therefore, SHH blockade seems to be a promising approach in preventing metastasis, which simultaneously increases intratumoral drug concentrations in fibroblast-enriched pancreatic cancer^4,15,16^. GDC-0449 (vismodegib) is a commercial hedgehog inhibitor, and several combination therapies comprising GDC-0449 and chemotherapeutics are currently in clinical trials for pancreatic cancer (NCT01088815, NCT00878163).

Doxorubicin (Dox) is an anthracycline antibiotic and a first-line anti-neoplastic drug for the treatment of a wide variety of cancers^17^. The main side effect of Dox is cardiotoxicity, which can be drastically reduced by encapsulation in a polyethylene glycol (PEG)-coated liposome or by other nano-formulations^18^. However, Dox has low response rates in pancreatic cancer when used as a single agent because of acquired resistance^19^ and limited penetration^19–21^. In the present study, the potential therapeutic strategy of a combination of nano-sized PEG-PCL-Dox micelles and GDC-0449 was investigated in pancreatic cancer *in vitro* and *in vivo*. Co-culture models employing pancreatic cancer cell lines and fibroblasts provide a useful platform because of its fibroblast-enriched microenvironment, which allows the investigation of potential drug combinations.

## Results

### GDC-0449 does not act synergistically with Dox in monocultured tumor cells

**Figure 1:**
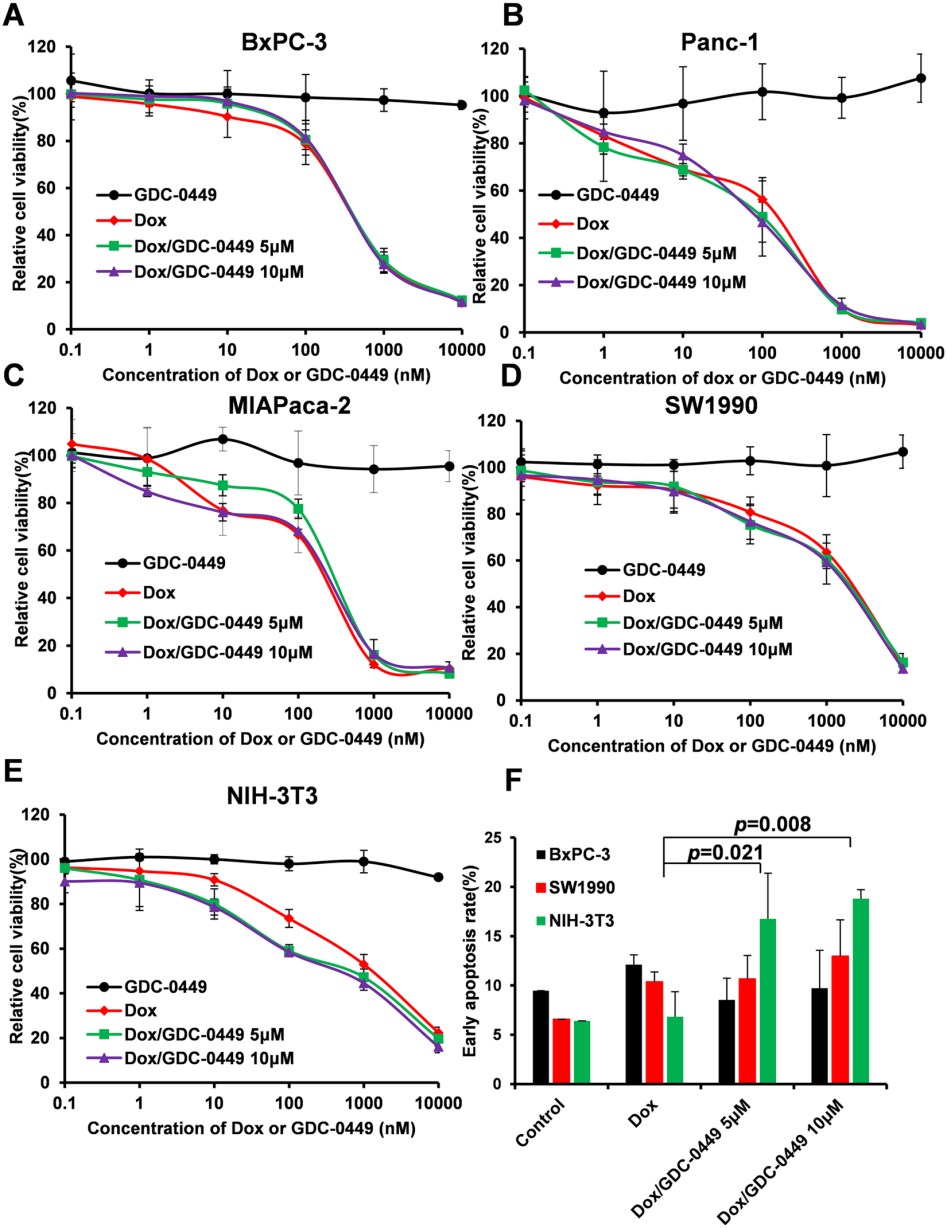
GDC-0449 improves the anti-proliferation activity in NIH-3T3 cells but not in BxPC-3, Panc-1, MIAPaca-2 and SW1990 cells when cultured alone. The cytotoxicities of GDC-0449, Dox or Dox with GDC-0449 (5 μM or 10 μM) were measured by performing a 48-h MTT assay in BxPC-3 (**A**), Panc-1 (**B**), MIAPaca-2 (**C**), SW1990 (**D**) and NIH-3T3 (**E**) cells. Apoptosis rate was detected by flow cytometry (Annexin V staining) analysis after treatment with Dox (200 nM) with or without GDC-0449 for 24 h (**F**). Data are presented as the mean ± SD. *P < 0.05, **P < 0.01.

To examine the cytotoxicity of GDC-0449, Dox, and the Dox/GDC-0449 combination, a 48-h MTT assay was performed in pancreatic cancer cell lines (BxPC-3, SW1990, Panc-1, and MIAPaca-2) and a non-transformed fibroblast cell line (NIH-3T3). Figure 1 shows that no obvious cytotoxicity was observed for GDC-0449 treatment in all cell lines. The combination of GDC-0449 and Dox did not show benefits over a single treatment of Dox in tumor cells (Fig. 1A–D). In contrast, the combination presented improved cytotoxicity in NIH-3T3 cells (Fig. 1E). Similar results were also obtained by using an apoptotic assay. GDC-0449 increased the apoptotic rate from 6.82% (Dox alone) to 16.8% (Dox + 5 μM GDC-0449) and 18.8% (Dox + 10 μM GDC-0449) in NIH-3T3 cells, but not in BxPC-3 or SW1990 cells (Fig. 1F).

### GDC-0449 can reverse fibroblast-induced Dox resistance of SMO-positive pancreatic cancer cells

**Figure 2:**
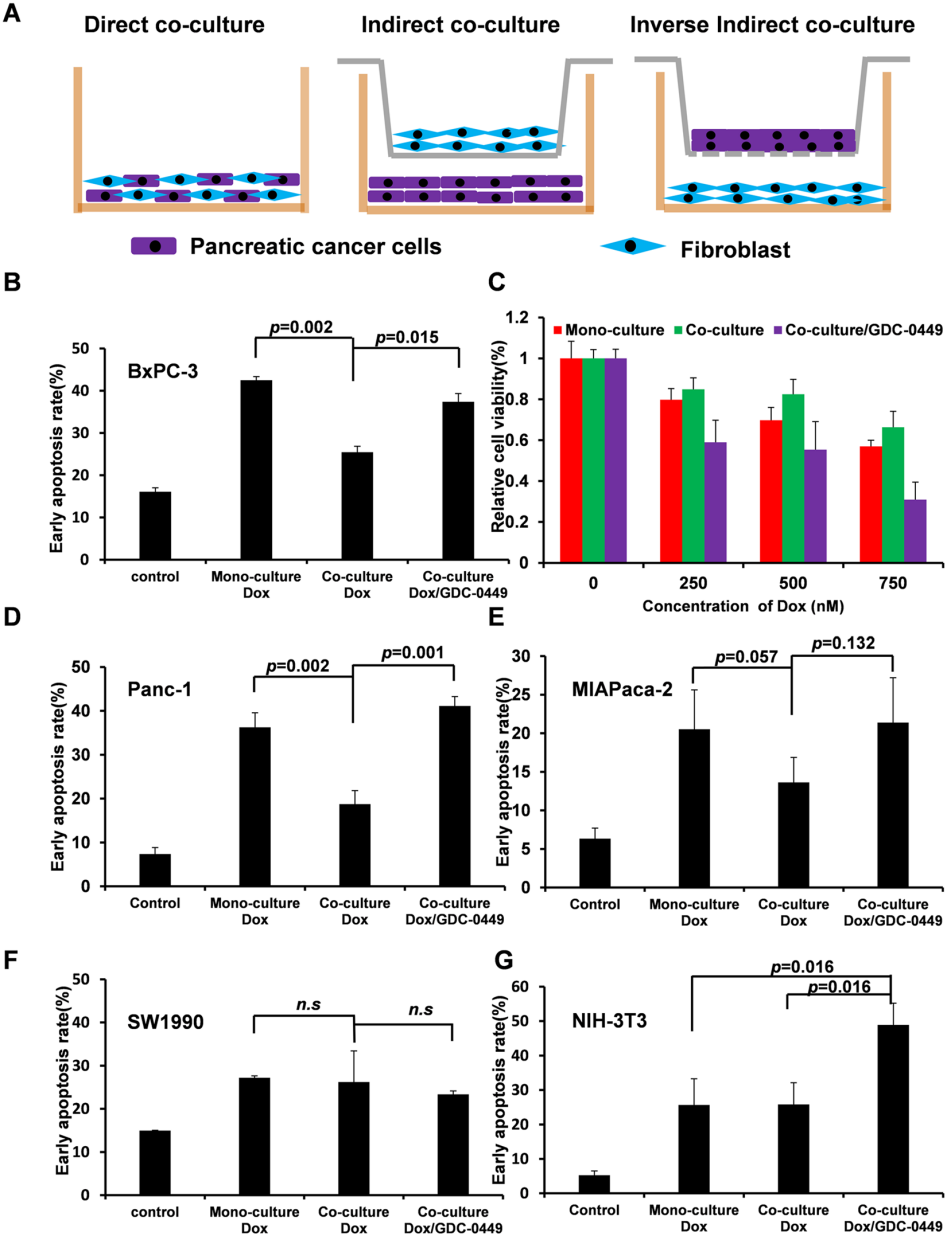
GDC-0449 reversed co-culture induced Dox resistance in SMO positive (BxPC-3, Panc-1, MIAPaca-2) cells, but not in SW1990 cells. Illustration of the direct co-culture and indirect co-culture models and inverse co-culture model for mechanism studies (**A**). In the indirect co-culture model, apoptotic BxPC-3 (**B**), Panc-1(**D**), MIAPaca-2 (**E**), and SW1990 (**F**) cells were detected by flow cytometry using Annexin V staining after the treatment of Dox (500 nM) with or without GDC-0449 (10 μM) for 24 h. In the direct co-culture model, luc^+^ BxPC-3 cells were co-cultured with luc-NIH-3T3 cells, and the viability of BxPC-3 cells was measured based on luciferase expression (**C**). In the inverse indirect co-culture model, NIH-3T3 cells were co-cultured with BxPC-3 cells, and apoptotic NIH-3T3 cells were detected by flow cytometry after treatment with Dox (500 nM) with or without GDC-0449 (10 μM) for 24 h. Data are presented as the mean ± SD. *P < 0.05, **P < 0.01.

To investigate fibroblast-induced drug resistance in the tumor microenvironment, we co-cultured tumor cells with NIH-3T3 fibroblasts directly or indirectly and evaluated the effect of Dox treatment with or without GDC-0449 (Fig. 2). In the transwell^®^-based indirect co-culture model, co-culturing with NIH-3T3 cells decreased the percentage of apoptotic BxPC-3 cells from 42.5% to 25.4% (*p* = 0.002), and GDC-0449 recovered the sensitivity of BxPC-3 cells to Dox (apoptotic rate = 37.4%) (Fig. 2B). Similar results were also obtained by measuring the light output of the luciferase enzyme in the direct co-culture system, where BxPC-3 luciferase-positive cells were seeded together with luciferase-negative NIH-3T3 cells (Fig. 2C). For BxPC-3 cells that were not *KRAS* mutants, we performed experiments on another two pancreatic cancer cell lines, MIAPaca-2 and Panc-1 with *KRAS* mutant^22^ and SMO-positive^14^, respectively (Fig. 2D,E). Similar to that observed in BxPC-3 cells, co-culturing with NIH-3T3 induced Dox resistance in these two cell lines, which was then reversed by GDC-0449.

In contrast, the fibroblast-induced Dox resistance and the effect of GDC-0449 were not observed when Smo protein-negative SW1990 cells were co-cultured with NIH-3T3 cells (Fig. 2F). Because NIH-3T3 cells exhibited the most dramatic response to Dox/GDC-0449 in a monoculture, we designed an inverse indirect co-culture experiment (Fig. 2A) to observe the effects of Dox and GDC-0449 on NIH-3T3 cells when grown as a co-culture (Fig. 2G). In contrast to tumor cells that obtained resistance to Dox when grown together with fibroblasts, NIH-3T3 cells did not develop resistance to Dox when grown as a co-culture. In addition, the synergistic cytotoxicity of GDC-0449 and Dox in NIH-3T3 cells was not dependent on co-culturing.

### GDC-0449 inhibits the fibroblast-induced upregulation of SHH signaling-related proteins in BxPC-3 cells

**Figure 3:**
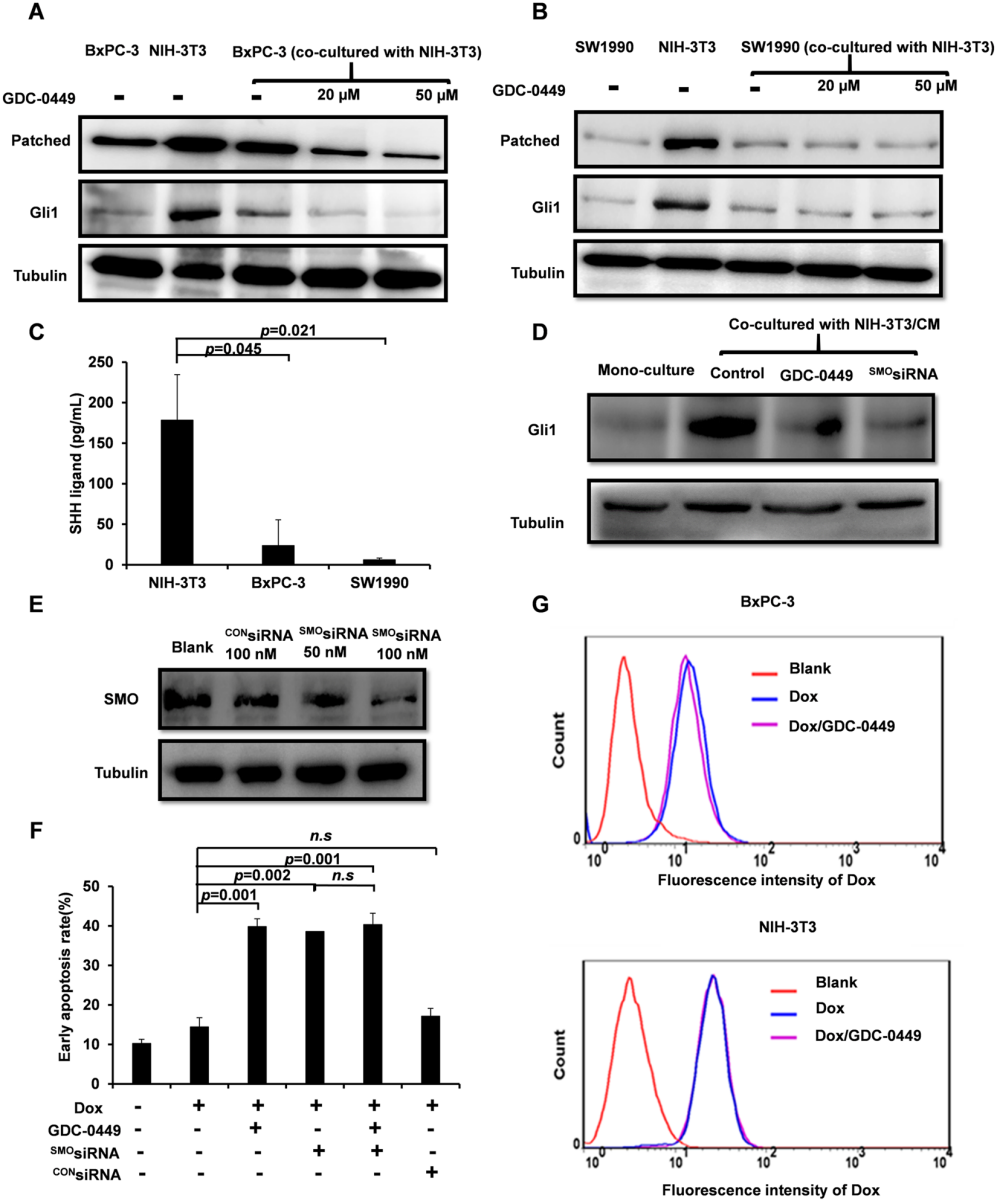
GDC-0449 reversed the fibroblast induced drug resistant through SHH inhibition. (**A**,**B**) The expression of SHH related proteins Gli1 and Patched in BxPC-3 (**A**) and SW1990 (**B**) cells when cells were co-cultured with NIH-3T3 through the transwell-based co-culture system. (**C**) The amount of SHH ligands in NIH-3T3/CM, BxPC-3/CM or SW1990/CM measured by the SHH ELISA kit. (**D**) The expression of Gli1 protein after different treatments in BxPC-3 cells. The concentration of GDC-0449 was 10 μM. (**E**) The expression of SMO protein in BxPC-3 cells when cells were successively transfected with ^CON^siRNA (100 nM) or ^SMO^SiRNA (50 nM or 100 nM). (**F**) GDC-0449 reversed Dox resistance which is induced by fibroblasts through SHH inhibition. (**G**) The effect of GDC-0449 on the cellular internalization of Dox (5 μg/mL) in BxPC-3 and NIH-3T3 cells. Data are presented as mean ± SD. *P < 0.05, **P < 0.01.

To elucidate the molecular mechanisms of GDC-0449 in a fibroblast-involved tumor microenvironment, Western blot analysis was performed in indirectly co-cultured tumor cells *in vitro* and in tumor samples *in vivo*. Figure 3 shows that NIH-3T3 cells expressed a significantly higher level of SHH signaling-related proteins Patched and Gli1 compared to the BxPC-3 and SW1990 cells. When BxPC-3 cells were co-cultured with NIH-3T3, the expression of GLi1 and Patched were significantly upregulated and GDC-0449 inhibited the co-culture-induced upregulation in a dose-dependent manner (Fig. 3A). However, the expression profiles of GLi1 and Patched in SW1990 cells was generally the same after co-culture with NIH-3T3 cells, and no significant effect was observed after GDC-0449 treatment (Fig. 3B).

To find out the main source of SHH ligands, the concentrations of secreted SHH ligands in different conditional media (CM) were measured by a SHH ELISA kit. Figure 3C shows that the amount of SHH ligand was 178 pg/mL in NIH-3T3/CM, which is 7.5 and 28.8 times higher than that in BxPC-3/CM and SW1990/CM, respectively. Meanwhile, the expression of Gli1 in BxPC-3 cells significantly increased when treated with NIH-3T3/CM and GDC-0449 reduced Gli1 in the similar profile to SHH silencing by ^SMO^siRNA (Fig. 3D). Western blots observations revealed that the SMO-specific siRNA reduced SMO protein expression in BxPC-3 cells (Fig. 3E). Importantly, in the co-culture system, GDC-0449 cannot further increase the cytotoxicity of Dox when SMO was knocked down in the BxPC-3 cells (Fig. 3F), indicating SHH dependent synergistic effect of GDC-0449 to Dox. In addition, the cellular uptake of Dox in BxPC-3 and NIH-3T3 cells was not affected by GDC-0449 treatment (Fig. 3G).

### GDC-0449 improves the antitumor activity of PEG-PCL-Dox

**Figure 4:**
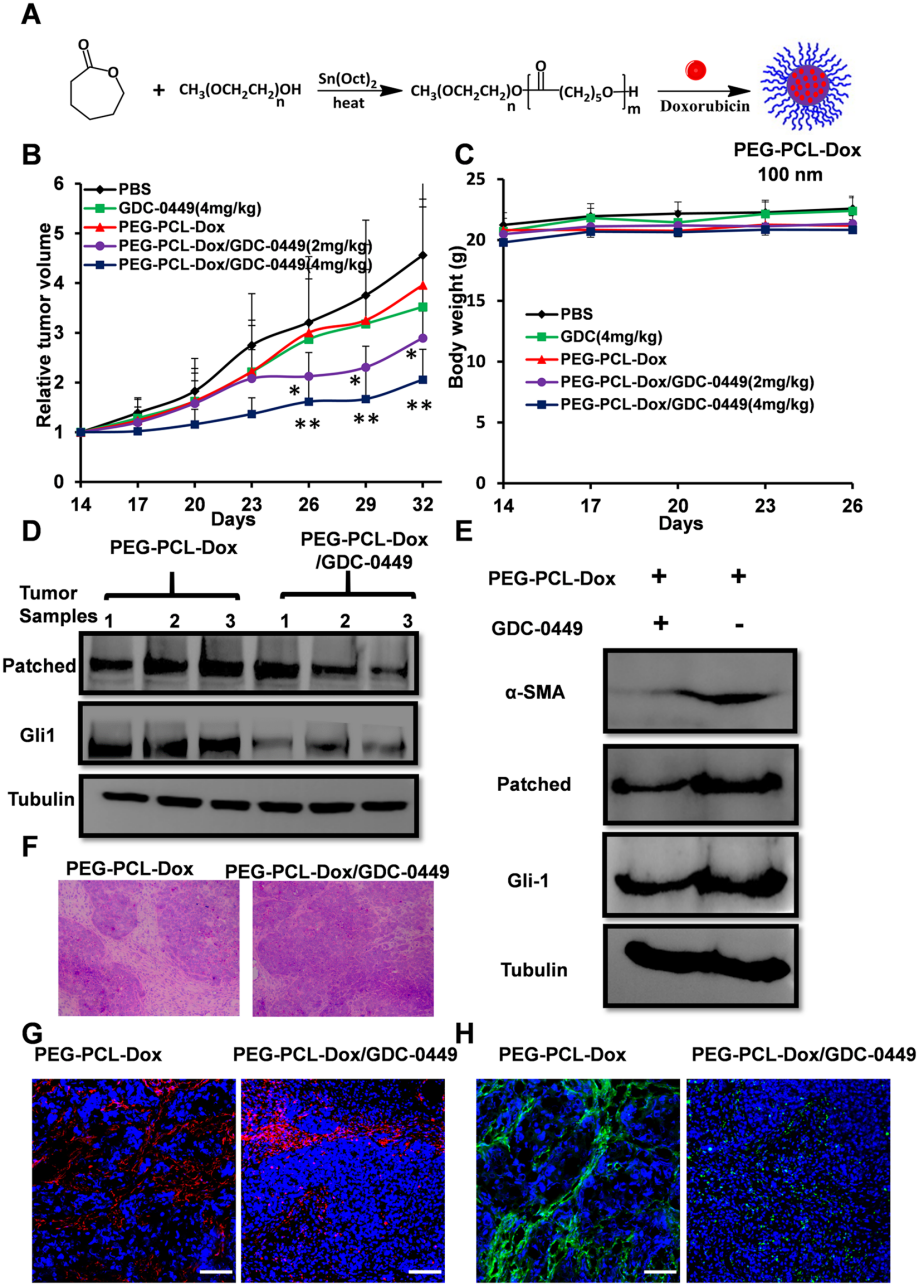
GDC-0449 improved the antitumor activity of PEG-PCL-Dox in BxPC-3 tumor xenografts. (**A**) Preparation of PEG-PCL and PEG-PCL-Dox micelles; (**B**) The relative tumor volume of each group with a function of time; (**C**) Body weight of mice in each group during treatment; (**D**) The expression of SHH marker GLi1 and Patched in tumors from mice treated with PEG-PCL-Dox or PEG-PCL-Dox/GDC-0449 (4 mg/kg). (**E**) The expression of α-SMA, Gli1, and Patched were downregulated by GDC-0449. Mice were randomly chosen from the PEG-PCL-Dox or PEG-PCL-Dox/GDC-0449 (4 mg/kg) group. (**F**) Representative H&E stained sections of tumors from the PEG-PCL-Dox group or PEG-PCL-Dox/GDC-0449 (4 mg/kg) group resected at 24 h after the 6^th^ injection; (**G**,**H**) Immunofluorescence staining of tumor-associated fibroblast markers, vimentin (**G**) and α-SMA (**H**) on tumor tissues from the PEG-PCL-Dox group or PEG-PCL-Dox/GDC-0449 (4 mg/kg) group, respectively. The red color represents vimentin, α-SMA is indicated in green, and the cell nuclei are stained blue. Scale bars = 100 μm. Data are presented as the mean ± SD. *P < 0.05, **P < 0.01.

PEG-PCL-Dox micelles with an average size about ~100 nm were prepared to prolong its circulation in blood and to reduce the side effects of free Dox (Fig. 4A). Nude mice bearing BxPC-3 xenografts were used to evaluate the synergistic effect of GDC-0449 on the nano-formulation of Dox. Figure 4B shows that PEG-PCL-Dox (4 mg/kg) or GDC-0449 (4 mg/kg) treatment alone imparted limited anti-tumor effects, and no significant difference in the average volume of tumors from that using PBS was observed. Importantly, tumor growth was remarkably inhibited when the combination therapy PEG-PCL-Dox/GDC-0449 was administered to the mice. Meanwhile, a dose-dependent and synergistic effect of GDC-0449 and PEG-PCL-Dox was observed as a higher dose of GDC-0449 (4 mg/mL) further improved the anti-tumor activity of PEG-PCL-Dox. In addition, no weight loss was observed in any treatment group (Fig. 4C). We examined the expression of Gli1and Patched regulated by SHH signaling in tumor samples after treatment in the presence or absence of GDC-0449. The results by Western blot analysis were shown on Fig. 4D. Patched was decreased in 2/3 of the tumor samples, while Gli1 was decreased in all 3 sample s, indicating GDC-0449 downregulated the SHH signaling *in vivo* (Fig. 4D,E).

H&E staining showed that compared with the PEG-PCL-Dox treatment, the administration of the combination therapy resulted in a significant reduction in the density of tumor cells (Fig. 4F). PDAC is characterized by abundant stroma that consists of activate tumor-associated fibroblasts that express vimentin and α smooth muscle actin (α-SMA). Vimentin and α-SMA can thus be utilized as biomarkers as well as prognosticators of pancreatic cancer^23^. In the present study, we examined the expression of vimentin and α-SMA in the subcutaneous BxPC-3 xenograft tumors that was treated with PEG-PCL-Dox with or without GDC-0449 (4 mg/kg). Representative immunostaining images (Fig. 4G,H) indicated that GDC-0449 significantly reduced the expression of vimentin and α-SMA. The changes in expression levels of α-SMA were further confirmed by Western blot analysis (Fig. 4E).

## Discussion

Pancreatic cancer is a deadly disease largely because of its propensity to rapidly undergo metastasis, coupled with the absence of an effective treatment due to the dense stroma that protects and supports the tumor^24,25^. Tumor-activated fibroblasts in pancreatic cancer are a key source of cytokines that aid tumor-stroma crosstalk and are strongly associated with pancreatic cancer progression by facilitating metastasis^26^, chemoresistance^27^, and restricting drug delivery^4,28^. Another key feature of pancreatic cancer is the abnormal activation of SHH signaling, which occurs in 70% of patients^29^. The four pancreatic cancer cell lines used in the present study, namely, BxPC-3, Panc-1, MIAPaca-2 (SMO-positive), and SW1990 (SMO-negative), have different SMO expression profiles^10,30^. In addition, the non-transformed NIH-3T3 mouse embryonic fibroblasts have been extensively utilized in investigations on tumor-stroma interactions in the microenvironment^31–33^ as well as SHH signaling^34^. Two independent research groups respectively led by Deuel and Huang reported that co-culturing with NIH-3T3 accelerates tumor cell growth *in vitro and in vivo*. Xenografts of tumor cells with NIH-3T3 cells facilitate in the development of a stroma-rich model that mimics the structure of human bladder cancer isolates^35^. Thus, NIH-3T3 cells were selected in the present study to investigate the effects of fibroblasts on the tumor microenvironment.

**Figure 5:**
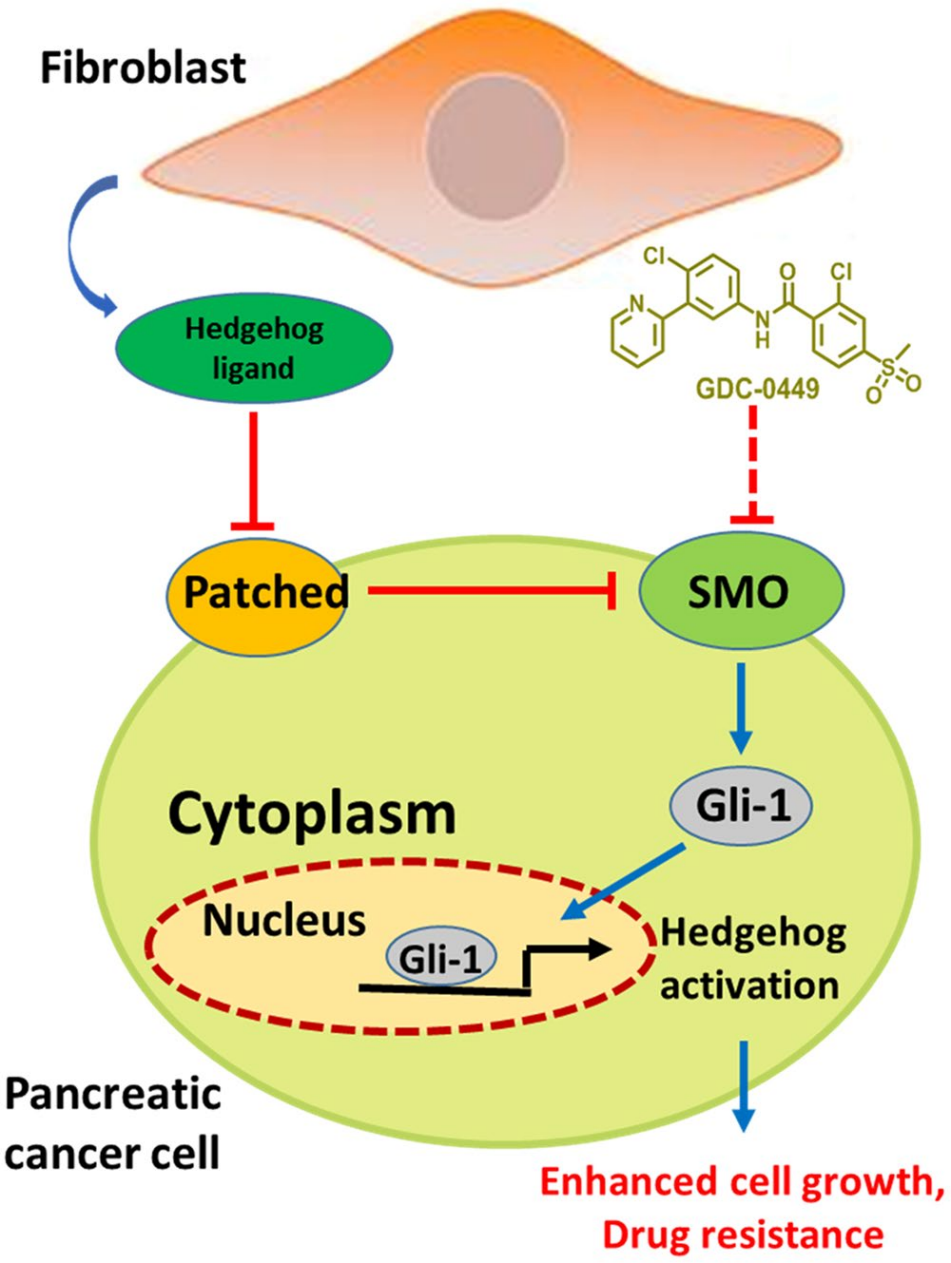
Schematic overview of the synergistic mechanisms of GDC-0449 with Dox in fibroblast-enriched pancreatic cancer. SHH signaling is activated in pancreatic cancer cells through the interaction between tumor cells and fibroblasts in the microenvironment. The aberrant activated SHH signaling promotes tumor growth and drug resistance in tumor cells, which can be revered by a commercial SMO antagonist and hedgehog inhibitor GDC-0449.

GDC-0449 inhibits SHH signaling by antagonizing SMO, which triggers the induction of SHH target genes^36^. In the present study, GDC-0449 was relatively non-toxic to all the five cell lines and did not exhibit synergistic effects with Dox on monocultured BxPC-3, Panc-1, MIAPaca-2, and SW1990 pancreatic cancer cells, thereby indicating that the proliferation of these cell lines is not dependent on SHH signaling. However, GDC-0449 improved the anti-tumor activity of Dox in SMO-positive tumor cells in a fibroblast-enriched microenvironment both *in vitro* and *in vivo*. As shown in Fig. 5, these findings are not only consistent with previous reports that have shown that tumor stroma could be the source of SHH ligands^12,37^ and active SHH signaling in tumors, but also reveal a SHH-related mechanism of resistance to Dox in pancreatic cancer. It was reported that GDC-0449 could be an inhibitor of ATP-binding cassette (ABC) transporters^38^. In this study, we ruled out this non-hedgehog pathway related effects of GDC-0449 through detecting the effect of GDC-0449 on cellular internalization of Dox and measuring the synergistic effect of GDC-0449 with Dox after silencing of SMO.

In a clinical trial of metastatic pancreatic cancer, the combination of GDC-0449 and gemcitabine was not superior to gemcitabine treatment alone^21^. Recently, several independent studies also reported that SHH signaling restrained pancreatic cancer progression^39–42^. A previous study has shown that nanodrugs are effective in the treatment of pancreatic cancer. The addition of albumin-bound paclitaxel (nab-paclitaxel) to standard gemcitabine treatment increased the response rate, progression-free survival, and overall survival of patients with metastatic pancreatic adenocarcinoma^40^. An interim report of a phase Π study indicated that GDC-0449 further improves the overall survival in the combination therapy with gemcitabine plus nab-paclitaxel^35,43^. The discrepancy in the findings may reflect the time-dependent role of stromal fibroblasts^44^ and indicate that the benefit of SHH signaling inhibition would depend on a diverse combination of treatments. Here, we report the synergistic effect of GDC-0449 and Dox only in SMO-positive cells (BxPC-3, Panc-1, and MIAPaca-2), but not in SMO-negative SW1990 cells, thereby suggesting that the effect of GDC-0449 also relied on SMO expression in tumor cells of individual patients.

Nanomedicines are apparently more beneficial in the treatment of pancreatic cancer compared to small molecules, and several nanodrugs have been approved by FDA in combination therapy for advanced pancreatic cancer such as albumin-bound paclitaxel (Abraxane) and liposomal irinotecan (Onivyde). Liposomal Dox (Doxil) combined with other drugs are now in clinical trials for advanced cancers, including pancreatic cancer (NCT02331251 and NCT02262455). Compared to free Dox, the nano-formulation of Dox reduces its cardiotoxicity but does not improve its therapeutic efficacy, which is largely due to the tumor heterogeneity in patients^3,45^. Similar to a previous report^20^, the 100-nm PEG-PCL-Dox showed no obvious anti-tumor activity against BxPC-3 tumors as a single treatment. In the low vascularized BxPC-3 tumor xenograft model, tumor nests are surrounded by thick fibrotic tissue^20,46^, and fibroblast-induced drug resistance and limited penetration could result in the poor therapeutic outcomes. Importantly, the GDC-0449/PEG-PCL-Dox combination inhibits BxPC-3 tumor growth synergistically, which could result from the reduced tumor density and downregulation of SHH signaling observed in tumor tissues.

In conclusion, our study provides a novel and potential treatment paradigm for fibroblast-enriched pancreatic cancer using the combination of nano-Dox and SHH signaling inhibitor GDC-0449. We also suggest that the expression status of SMO in tumors be considered prior to the administration of GDC-0449 as part of a treatment regimen for pancreatic cancer.

## Methods

### Materials, cell lines and animals

GDC-0449 was purchased from Selleck (Shanghai, China) and doxorubicin hydrochloride (≥99%) was obtained from Zhejiang Hisun Pharmaceutical Company. ^SMO^siRNA duplexes (sense 5′-UGCCCAAGUGUGAGAAUGAUU-3′) were purchased from GenePhama (Shanghai, China) and non-targeting siRNA duplexes served as controls. Human primary pancreatic adenocarcinoma cell lines BxPC-3, SW1990, Panc-1, MIAPaca-2 and mouse embryonic fibroblast cell line NIH-3T3 were obtained from the cell bank of Chinese Academy of Sciences (Shanghai, China). BALB/C female nude mice, 6–8 weeks of age were purchased from the institute of medicine, Zhejiang province, and housed in sterile cages with a standard condition. All work performed on animals were approved by the Animal care and Use Committee of Zhejiang University and all methods were performed in accordance with the relevant guidelines and regulations.

### Synthesis of PEG-PCL-Dox

PEG_5000_-PCL_5000_ and Dox-loaded PEG-PCL micelles were prepared as reported previously^47^. In brief, a series of PEG-PCL block copolymers were prepared through ring-opening polymerization of ε-caprolactone (ε-CL) initiated by mPEG_5000_ using stannous octoate as catalyst. The synthesized PEG-PCL was purified by precipitation for three times using diethyl ether and characterized by^1^H-NMR and GPC. Dox was loaded into PEG-PCL nanoparticles by the emulsion ultrasonic method, and free Dox was removed by centrifugation and dialysis. The size distribution of the Dox-loaded PEG-PCL micelles was measured on a Zetasizer Nano-ZS (Malvern Instruments, UK) and the amount of Dox loaded in the micelles was determined by HPLC.

### Cell culture

Tumor cells and NIH-3T3 fibroblast cells were cultured in 75 cm^2^ culture flasks with 15 mL medium in a cell incubator with 5% CO_2_ at 37 °C. SW1990 and NIH-3T3 cells were maintained in RPMI-1640 medium (Genom Biological Technology Co. Ltd., Hangzhou, China). BxPC-3, Panc-1 and MIAPaca-2 cells were grown in Dulbecco’s modified eagle’s medium (DMEM) (Genom Biological Technology Co., Ltd., Hangzhou, China). Culture medium was changed every 2-3 days. When the confluence reached 70–80%, cells were spilt with 0.25% Trypsin–EDTA, assessed for viability (>95% viable) and then suspended for experimental use.

### Anti-proliferation assay

The cytotoxicity of GDC-0449 or Dox to tumor cell lines or fibroblast cells was measured by 3-(4, 5-dimethylthiazol-2-yl)-2, 5-diphenyltetrazolium bromide (MTT) assay. Briefly, BxPC-3, SW1990 and NIH-3T3 cells were seeded at 4000 per well in 96-well plates and cultured overnight, followed by addition of GDC-0449 and Dox at different concentrations. After 48 h, 20 μL MTT reagent (sigma, dissolved in PBS, 5 mg/mL) was added into each well and cells were incubated for another 4 h. Finally, MTT was removed and 150 μL DMSO was added. The absorbance in each well was determined at 562/620 nm using a Molecular Devices microplate reader according to the manufacturer’s instructions. Each measurement was performed in triplicate and repeated in three independent experiments.

### Detection of apoptosis

Cells were first incubated with GDC-0449 (10 μM) for 24 h, and then incubated with free Dox for another 24 h. After incubation, cells were harvested and stained with FITC-labeled annexin V for 15 min in the dark. Early apoptotic cells were detected by flow cytometry (Becton Dickinson, FACSCalibur™, San Jose, USA).

### Cell co-culture

Direct co-culture: Stably transfected luciferase positive BxPC-3 cells were seeded with luciferase negative NIH-3T3 cells. The number of tumor cells was determined by the intensity of the bioluminescent signal. Indirect co-culture: The transwell (Corning, Cat.3452) co-culture system was used to establish the indirect co-culture model. NIH-3T3 cells were seeded on the inserts, and BxPC-3 or SW1990 cells were seeded on normal 6-well dishes. After 24 h, the inserts seeded with NIH-3T3 cells were moved to the upper compartment of the 6-well dishes. Treatments were performed after co-culture for 2 days. Each co-culture experiment was performed three times to validate the results

### Preparation of conditional medium of different cells

NIH-3T3 conditional medium (NIH-3T3/CM), BxPC-3/CM, SW1990/CM were prepared just as paper reported^48^. Briefly, when the density of NIH-3T3, BxPC-3 or SW1990 cells reached 70–80%, the medium was replaced to serum-free DMEM and cells were cultured for additional 24 h. The supernatant was then collected and ultrafiltered through an Amicon^®^ Ultra-4 3 K device. The amount of secreted SHH chemokine in the conditional medium was quantified by a SHH ELISA kit (Mlbio, Shanghai, China) according to the protocol provided by the supplier.

### Small interfering RNA silencing of SMO in BxPC-3 cells

BxPC-3 cells were seeded in a 6-well plate at a density of 2 × 10^5^ cells per well and transiently transfected with 100 or 50 nM ^SMO^siRNA or ^CON^siRNA using 5 μL lipofectamine 2000 (invitrogen) in a total transfection volume of 2 mL of FBS free MEM medium. After incubation at 37 °C for 4 h, the medium was replaced with 2 mL of fresh medium with 10% FBS and cells were cultured for another 48 h. Target suppression was assessed by Western blots analysis. The siRNA transfected BxPC-3 cells were co-cultured with NIH-3T3 cells for 48 h and then Dox was added and treated for another 24 h. Early apoptosis of BxPC-3 cells were detected as mentioned before.

### Cellular uptake of Dox

Cellular uptake profiles of Dox were measured by flow cytometry. In brief, BxPC-3 (2 × 10^5^) and NIH-3T3 (2 × 10^5^) cells were seeded into 12-well plates and incubated overnight. Cells were treated with GDC-0449 (10 μM) for 24 h, and then Dox (5 μg/mL) was added to each well and incubated for 6 h. The cells were then trypsinized, washed with cold PBS for three times, and resuspended in 0.4 mL PBS. Each sample was quickly analyzed on a flow cytometry.

### Western blot analysis

After treatments, tumor cells were washed twice with PBS and then lysed. Protein concentration of each sample was determined by Bradford assay. Cell lysates were separated by SDS-PAGE and transferred to a PVDF membrane, which was activated by methyl alcohol for five minutes before. The membrane was blocked for 1–2 h at room temperature with 5% non-fat milk, then washed with TBST buffer for three times and incubated overnight at 4 ^o^C with one of the following antibodies: Anti-Gli1 (Abcam ab49314, 1:1000), Anti-Patched (Abcam ab39266, 1:1000), Anti-Smoothened (Bioss bs-2801R) and anti-tubulin (Beyotime AT819, 1:1000). Chemiluminescence detection was performed with the corresponding second antibody conjugated with HRP. Images were acquired using BeyoECL plus and CLiNX Science instruments.

### Antitumor evaluation

Female BALB/C homozygous athymic nude mice, 6-8-week-old, were inoculated with BxPC-3 (2 × 10^6^ per mouse). When the average volume of tumors reached about 100 mm^3^ (V = ab^2^/2, a and b means the longest and widest diameter of tumor), mice were assigned to five treatment groups (n = 7): (1) PBS; (2) PEG-PCL-Dox (equivalent to 4 mg/kg Dox); (3) GDC-0449 (4 mg/kg, dissolved in 30% PEG-400 and 70% PBS); (4) PEG-PCL-Dox (4 mg/kg) and GDC-0449 (2 mg/kg); (5) PEG-PCL-Dox (4 mg/kg) and GDC-0449 (4 mg/kg). The treatment regimens were initiated on day 14 (*i.v*.), repeated every 3 days for 6 cycles. At the end of the experiment, mice were sacrificed according to institutional guidelines, and tumor tissues were resected, weighed, and divided into two parts, one for Western blot analysis and the other one was fixed in 10% neutral-buffered formalin for paraffin embedding.

### Analysis of tumor samples

Tumors were excised, homogenized and lysed using lysis buffer for half an hour on ice. Western blot analysis was performed as described previously to detect SHH signaling related proteins and α-SMA in tumor tissues. For H&E staining, tumor samples were washed in PBS, fixed with 4% neutral buffered paraformaldehyde, embedded in paraffin and cross-sectioned at a thickness of 10 μm. The sections were stained with hematoxylin-eosin (H&E, Beyotime, China) and observed under light microscopy.

### Immunofluorescence Assays

Immunofluorescence staining was performed as recommended by the manufacturer. Briefly, tumor sections with a thickness of 8 μm were fixed with cold acetone for 10 min, dried on slide rack for 30 min, rinsed with ttPBS for 3 times, blocked with 10% goat serum for 60 min at room temperature and then incubated with the primary antibody for α-SMA (Abcam ab32575, 1:100) and vimentin (Bioss, bs-0756R) at r.t. for 2 h. The sections were washed at least 3 times, at least 5 minutes each time in ttPBS and further incubated with FITC or Alexa Fluor 647 labeled goat anti rabbit second antibody (BD, 1:100) for 1 h at r.t. in the dark. Slides were rinsed with ttPBS and PBS for at least 3 times and the nuclei were stained with DAPI (Beyotime, C1006) staining buffer for 15 min. Fluorescent images were acquired by a confocal microscope (Nikon A1).

### Statistical analysis

Statistical analysis was performed using two-tailed, unpaired student’s t-test by Excel. *p* < 0.05 was considered statistically significant and all results were expressed as a mean ± standard deviation.

### Data availability

The authors declare that all data generated or analyzed during this study are included in this published article and its Supporting Information files or from the corresponding author upon request.

## Electronic supplementary material

Supporting information
