# Supplementary material for: GDC-0449 improves the antitumor activity of nano-doxorubicin in pancreatic cancer in a fibroblast-enriched microenvironment
Source: Sci Rep. 2017 Oct 17;7:13379. doi: 10.1038/s41598-017-13869-0 (PMC5645386; doi:10.1038/s41598-017-13869-0)
Supplement: Supplementary file 1 — Supporting information [file 41598_2017_13869_MOESM1_ESM.pdf]

Supporting information

GDC-0449 improves the antitumor activity of nano-doxorubicin in pancreatic cancer in fibroblast-enriched microenvironment

Quan Zhou, Yongcun Zhou, Xiangrui Liu*, Youqing Shen*

Key Laboratory of Biomass Chemical Engineering of Ministry of Education and Center for Bionanoengineering, College of Chemical and Biological Engineering, Zhejiang University, Hangzhou, China

*Corresponding authors E-mail address: xiangrui@zju.edu.cn, shenyq@zju.edu.cn

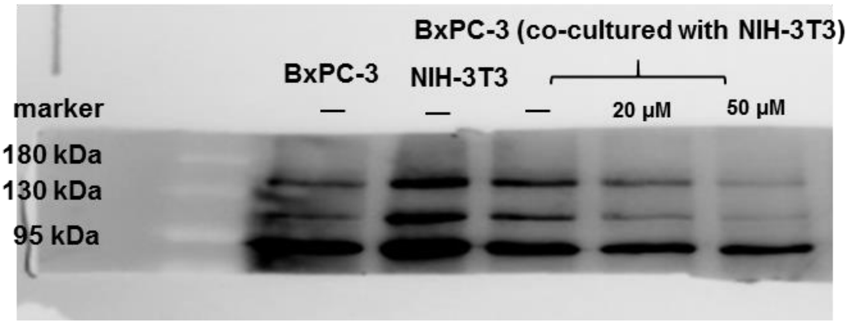

Fig. S1 Full-length blots of Gli1 in BxPC-3 cells

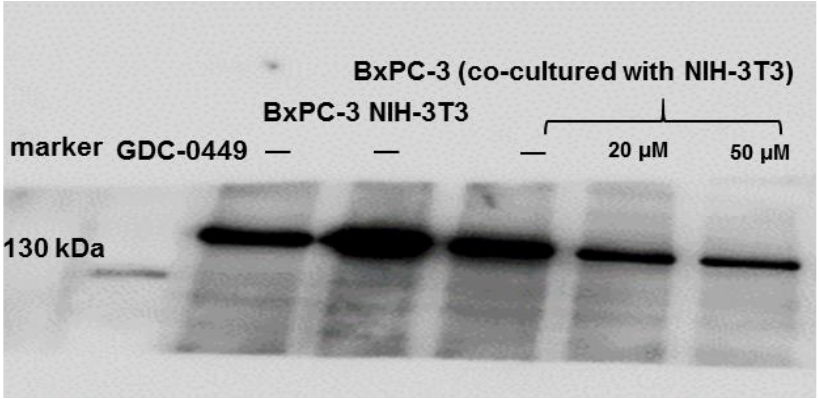

Fig. S2 Full-length blots of Patched in BxPC-3 cells

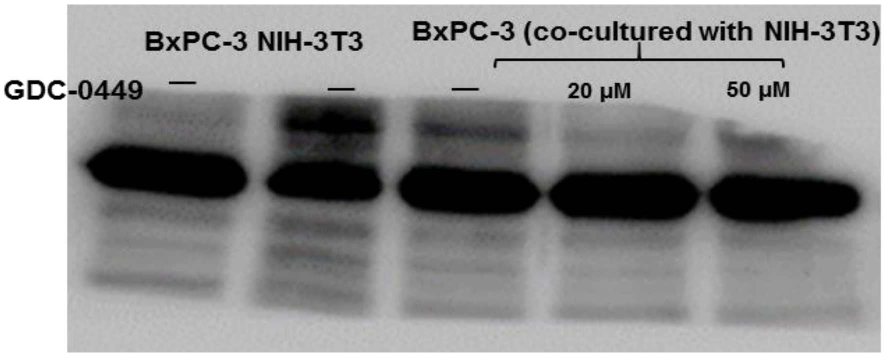

Fig. S3 Full length blots of Tubulin in BxPC-3 cells

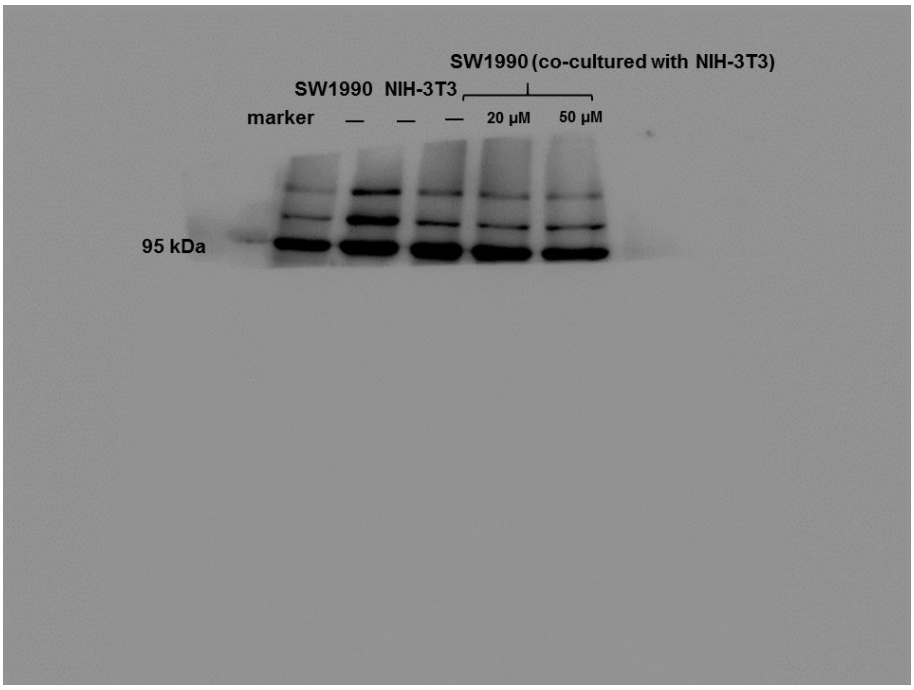

Fig. S4 Full-length blots of Gli1 in SW1990 cells

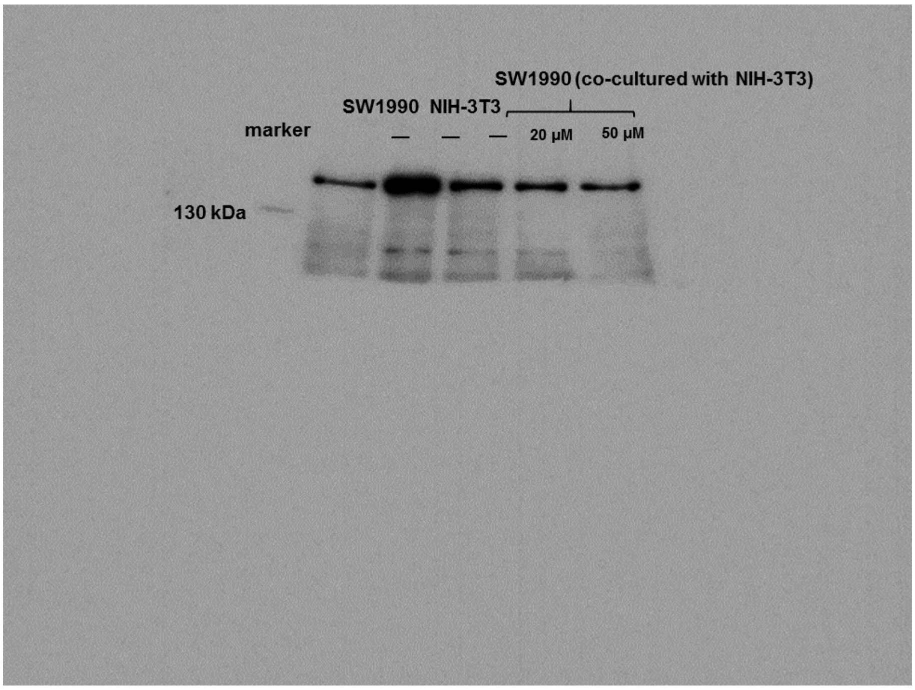

Fig. S5 Full-length blots of Patched in SW1990 cells

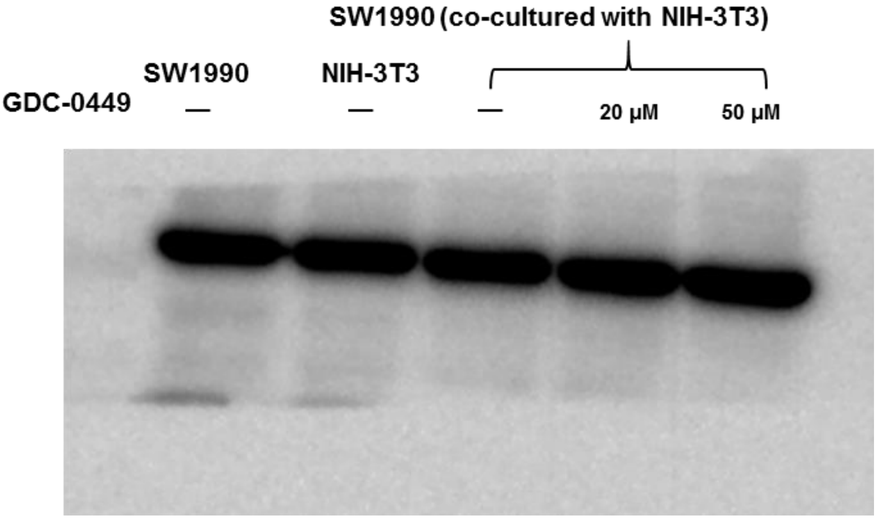

Fig. S6 Full-length blots of Tubulin in SW1990 cells

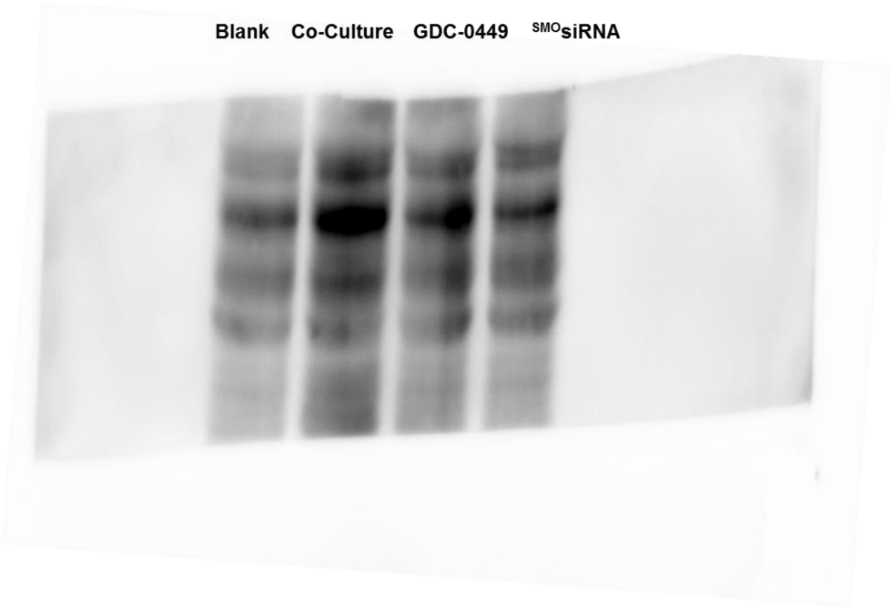

Fig. S7 Full-length blots of Gli1 in BxPC-3 cells co-cultured with NIH-3T3/CM

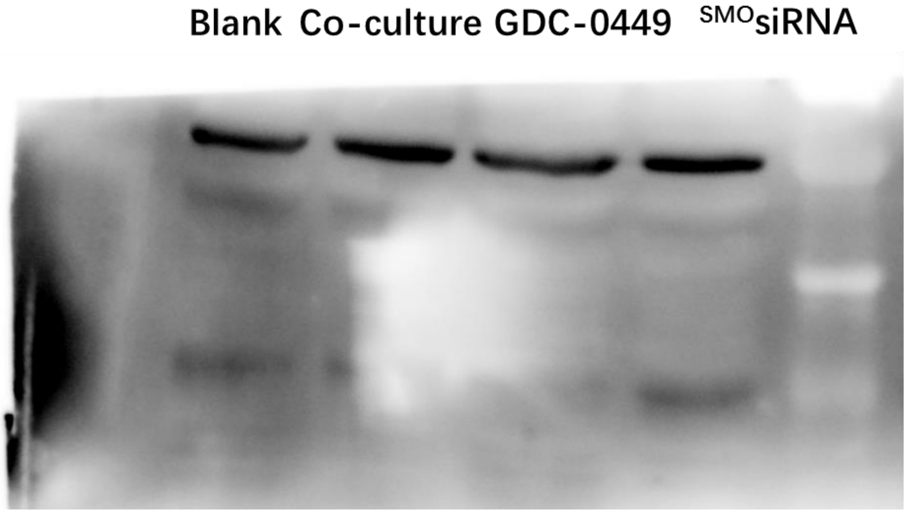

Fig. S8 Full-length blots of Tubulin in BxPC-3 cells co-cultured with NIH-3T3/CM

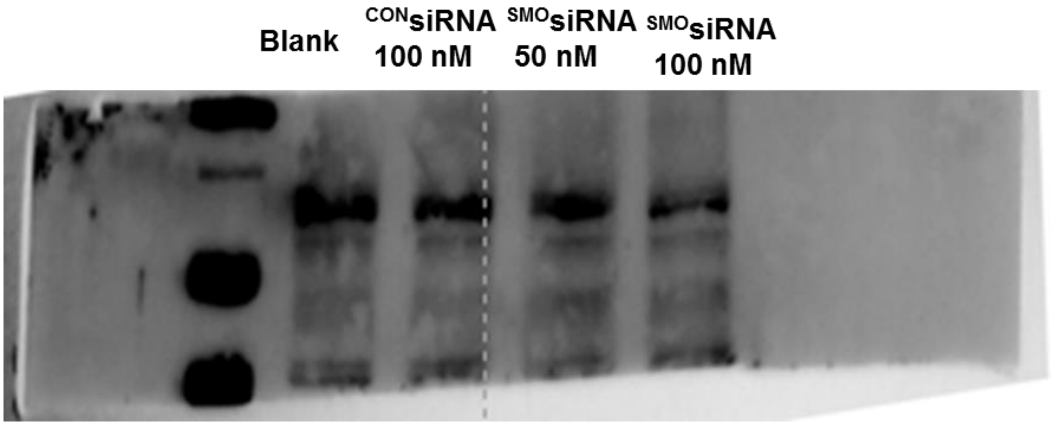

Fig. S9 Full length blots of SMO protein in BxPC-3 cells when cells were transfected with ^{CON}siRNA (100 nM) or ^{SMO}SiRNA (50 nM or 100 nM).

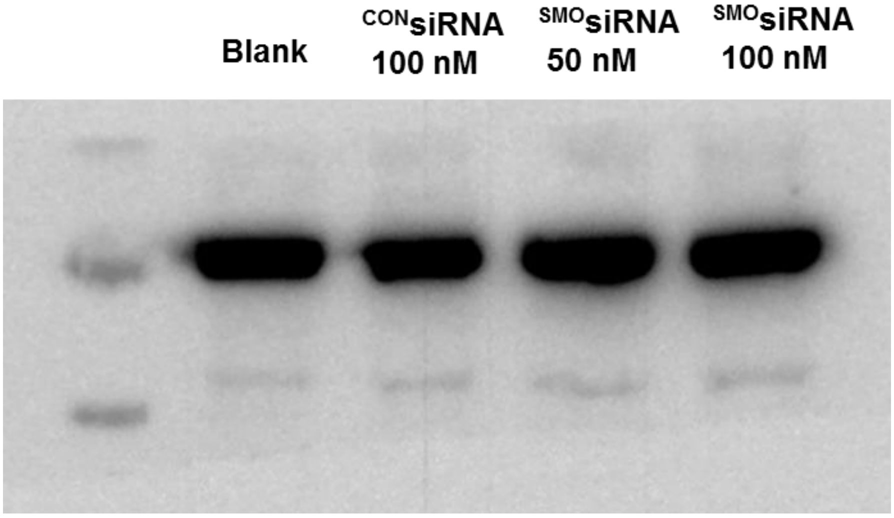

Fig. S10 Full length blots of Tubulin in BxPC-3 cells when cells were transfected with ^{CON}siRNA (100 nM) or ^{SMO}SiRNA (50 nM or 100 nM).

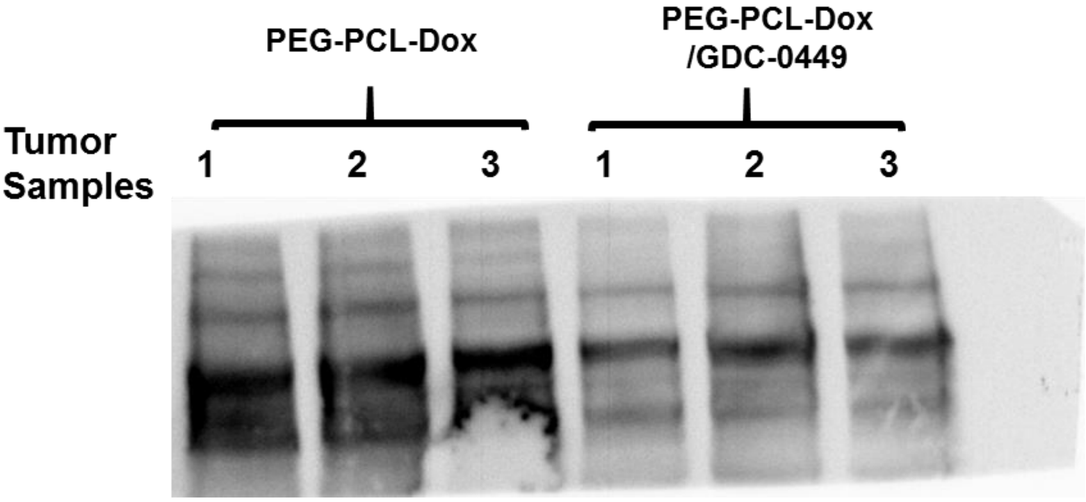

Fig. S11 Full-length blots of Gli1 in BxPC-3 xenograft tumor tissues

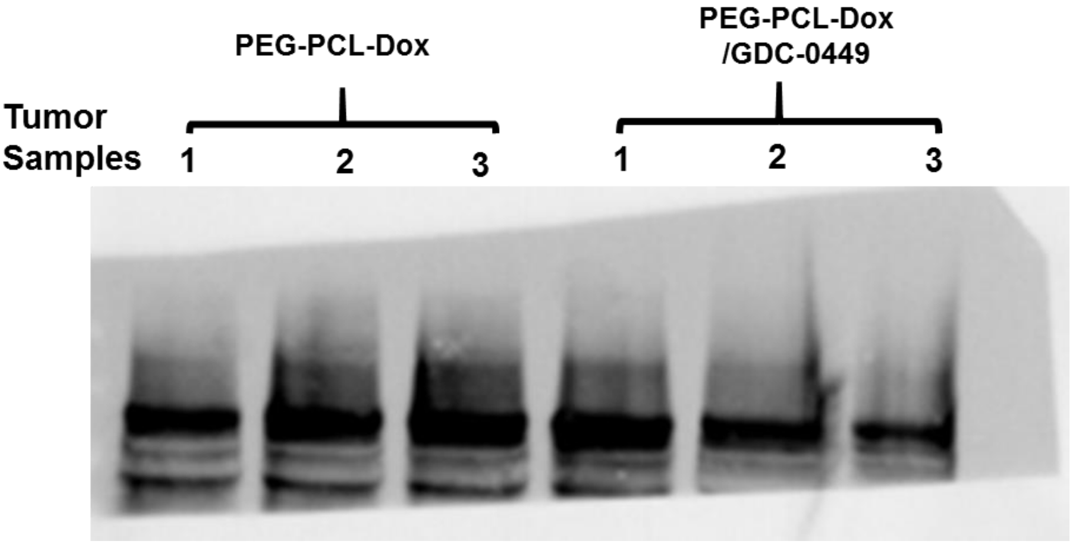

Fig. S12 Full-length blots of Patched in BxPC-3 xenograft tumor tissues

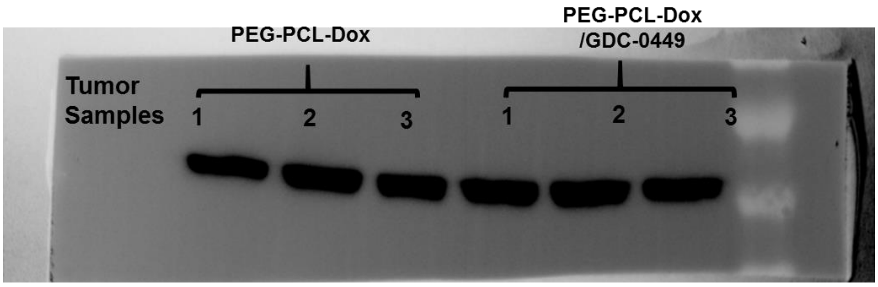

Fig. S13 Full-length blots of tubulin in BxPC-3 xenograft tumor tissues

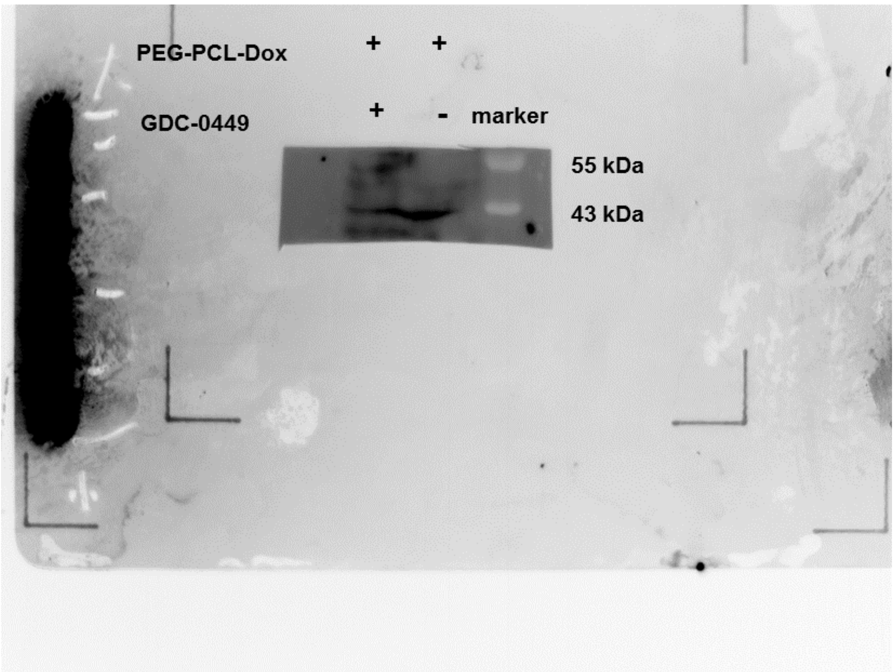

Fig. S14 Full-length blots of α-SMA in BxPC-3 xenograft tumor tissue

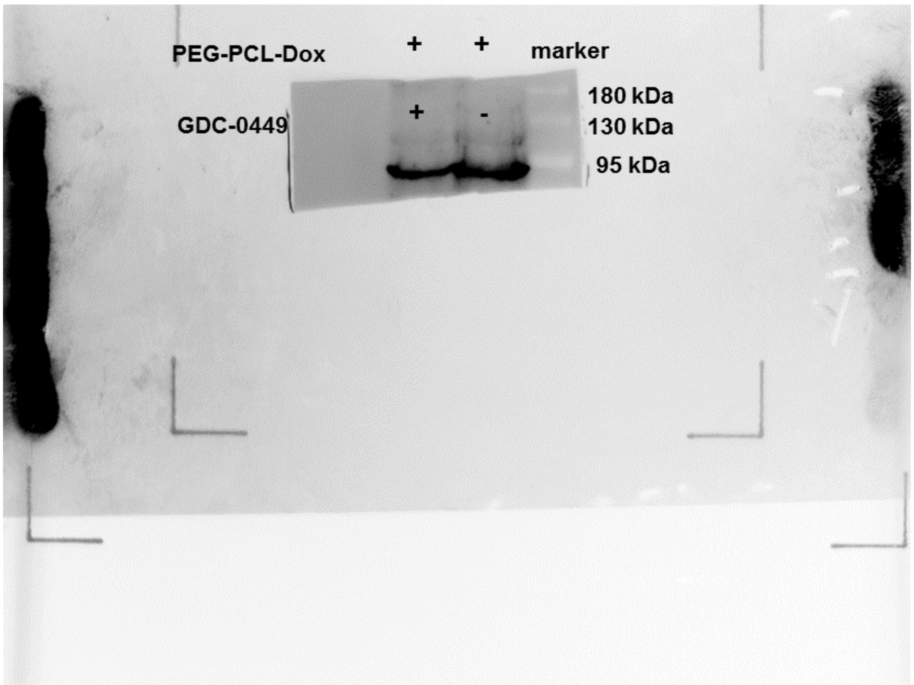

Fig. S15 Full-length blots of Gli1 in BxPC-3 xenograft tumor tissue

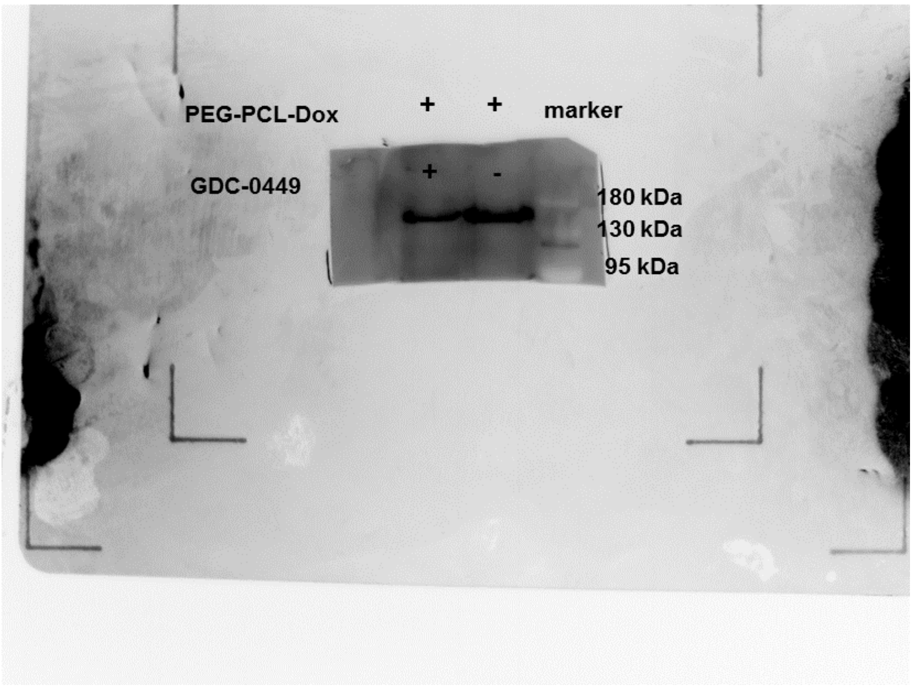

Fig. S16 Full-length blots of Patched in BxPC-3 xenograft tumor tissue

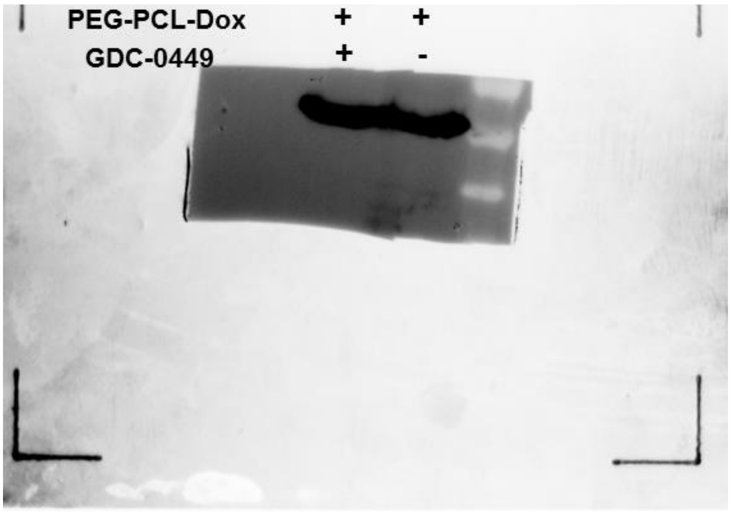

Fig. S17 Full-length blots of Tubulin in BxPC-3 xenograft tumor tissue
